# Questionnaire‐based epidemiological study of hidradenitis suppurativa in Japan revealing characteristics different from those in Western countries

**DOI:** 10.1111/1346-8138.15378

**Published:** 2020-05-22

**Authors:** Koremasa Hayama, Hideki Fujita, Takashi Hashimoto, Tadashi Terui, The Japanese HS Research Group

**Affiliations:** ^1^ Division of Cutaneous Science Department of Dermatology Nihon University School of Medicine Tokyo Japan; ^2^ Department of Dermatology Osaka City University Graduate School of Medicine Osaka Japan

**Keywords:** epidemiology, hidradenitis suppurativa, Hurley stage, modified Sartorius score, physician’s global assessment

## Abstract

Hidradenitis suppurativa (HS) is a chronic relapsing skin disease localized mainly on the apocrine gland‐bearing areas. In Japan, HS is yet to be fully understood, and no criteria have been established for its diagnosis or severity assessment. The purpose of this study was to investigate and characterize HS in Japan. We conducted a nationwide questionnaire‐based study, in which Japanese diagnostic criteria were proposed. Question items included age, sex, disease duration, past history, family history, smoking status, disease severity scores (Hurley stage, modified Sartorius score and Physician Global Assessment [PGA] score), treatments, comorbidities and prognosis. We analyzed 300 patients (219 males and 81 females) diagnosed with HS based on our criteria. Average disease duration was 92.3 ± 6.82 months. Only 12 (4%) patients had a family history of HS. Disease severity was classified by PGA score (mild, 100 [33.3%]; moderate, 133 [44.3%]; severe, 34 [11.3%]; most severe, 29 [9.7%]) and Hurley stage (I, 69 [23%]; II, 109 [36.3%]; III, 121 [40.3%]). Disease severities based on PGA score and Hurley stage were positively correlated to modified Sartorius score using the Kruskal–Wallis test (*P* < 0.001, respectively). Patients with diabetes mellitus showed higher PGA scores (χ^2^ = 10.977, *P* = 0.01185). Presence of axillary lesions related to higher PGA scores (χ^2^ = 8.6378, *P* = 0.03452). The results in this study and previous studies indicate that Japanese HS patients have different backgrounds from those in Western countries, and are characterized by male predominance, higher incidence of Hurley stages II and III, higher PGA scores in patients with axillary lesions and much fewer familial cases.

## Introduction

Hidradenitis suppurativa (HS) mostly affects the apocrine gland‐bearing areas of the body and shows a chronic relapsing course.^1^ However, the concept of HS is not well recognized in Japan, despite its significant impairment of patient quality of life (QoL). Moreover, because many Japanese HS patients sustain lesions on the buttocks,^2^ this condition has been called chronic pyoderma of the buttocks, which is categorized as an infectious skin disease. In addition, there were no diagnostic or severity assessment criteria. Although there is a small‐scale epidemiological study analyzing 100 Japanese HS patients,^2^ the features of HS in Japan are not fully understood. The purpose of this study was to investigate the actual picture of HS in Japan by conducting a nationwide questionnaire‐based epidemiological survey.

## Methods

Because there were no diagnostic criteria or severity classifications of HS in Japan, we prepared diagnostic criteria (Table 1) for this survey by referring to European criteria proposed in the second congress organized by the Hidradenitis Suppurativa Foundation.^3^ Although histopathology is not essential in the European criteria, histopathological elements were included in this study as diagnostic aids to confirm the diagnosis of HS by excluding other diseases, because the concept of HS is still not well‐recognized in Japan. A questionnaire‐based study was performed by sending questionnaires to the 670 hospitals/facilities that are providing dermatological training under the certification of the Japanese Dermatological Association. The propriety of the participation in this research and the patient number were verified by the first questionnaire. A secondary survey was then carried out at the participating facilities. The second questionnaire was sent to collect the data of patients who were diagnosed with HS based on the diagnostic criteria between 2012 and 2014. The question items in the second questionnaire included age, past history, family history, smoking, comorbidities, disease duration, severity (Physician Global Assessment [PGA] score as mild, moderate, severe and most severe; modified Sartorius score [MSS];^4^ and Hurley stage),^5^ treatment (antimicrobials, surgical resection, biologics, immunosuppressive agents and others) and carcinogenesis.

**Table 1:** Diagnostic criteria of hidradenitis suppurativa in this study

I: Diagnostic criteria: there are symptoms in one or more anatomical regions such as the axillae, groin, buttocks, anogenital area and others.
(1) Repeated appearance of abscess or drainage
(2) Scar or nodule
(3) Draining fistula
→If patients have exhibited in only one anatomical region, two or more out of three items ([1]–[3]) are required to be met. If patients have symptoms in two regions, one or more out of three items is/are required to be met for diagnosis.
*Infection and malignancy must be excluded.
II: Diagnostic aids (histopathological findings)
(1) Keratotic plug and infiltration of leukocytes in hair follicles
(2) Draining fistula or sinus tract in the dermis

This study was approved by the ethics committee of Nihon University Itabashi Hospital (RK‐150310‐11).

Statistical analyses were performed using R software (version 3.2.5), in which *P *≤ 0.05 was considered statistically significant. Severity was analyzed by Kruskal–Wallis test. The Steel–Dwass test was used for comparison between multiple groups. The χ^2^‐test was performed to evaluate the relationship between disease severity and the patient’s background.

## Results

The first questionnaire was sent to 670 institutions, 178 of which responded. Of these, 78 institutions agreed to participate in the study using a secondary questionnaire. The other respondents did not participate in the second survey because they had no HS patients. Twenty‐one institutions were not able to provide data because they did not follow the HS patients at the time of second survey. A total of 300 patients’ data from 57 institutions were collected for statistical analysis. All patients met the diagnostic criteria. One hundred and fifty‐seven patients (52.3%) received histopathological examination. Patient backgrounds are summarized in Table 2. Of the 300 patients, 219 (mean age, 44.5 ± 15.50 years) were male and 81 (mean age, 36.4 ± 13.63 years) female. The male : female ratio was 2.69: 1. The mean disease duration at first visit to each facility was 91.6 ± 6.82 months (7.58 ± 0.56 years). Only 12 patients (4%) had a family history.

**Table 2:** Backgrounds of the patients

No. of patients	
Total	300
Male	219 (mean age: 44.5 ± 15.50 years)
Female	81 (mean age: 36.4 ± 13.63 years)
Disease duration	91.0 ± 6.82 months (7.58 years)
Family history	
No	215 (71.7%)
Yes	12 (4.0%)
Unknown	73 (24.3%)
Past medical history	
Obesity	48 (16.0%)
Diabetes mellitus	55 (29.3%)
Hypertension	36 (12.0%)
Hyperlipidemia	19 (6.3%)
Crohn’s disease	1 (0.3%)
Hirsutism	17 (5.7%)
Smoking history	
No	85 (28.3%)
Yes	123 (41.0%)
Unknown	92 (30.7%)
Carcinogenesis	
Squamous cell carcinoma	1 (0.3%)
Histopathological examination	
No	143 (47.7%)
Yes	157 (53.3%)
Severity	
Physician Global Assessment	
Mild	100 (33.3%)
Moderate	133 (44.3%)
Severe	34 (11.3%)
Most severe	29 (9.7%)
Hurley stage	
I	69 (23.0%)
II	109 (36.3%)
III	121 (40.3%)

The past medical history of the patients included obesity in 48 (16%) of the 300 patients, diabetes mellitus in 55 (18.3%), hypertension in 36 (12%), hyperlipidemia in 19 (6.3%), Crohn’s disease in one (0.33%) and hirsutism in 17 (5.6%). Among them, only diabetes mellitus was correlated with higher PGA scores (χ^2^ = 10.977, *P* = 0.01185). One hundred and twenty‐three patients (41%) had a history of smoking, while 85 (28.3%) had no smoking history. Smoking histories were unknown for the remaining patients. There was no correlation between the MSS and smoking history (χ^2^ = 5.6894, *P* = 0.1277). One case (0.33%) with family history suffered from secondary squamous cell carcinoma (SCC) of the buttock.

**Figure 1:**
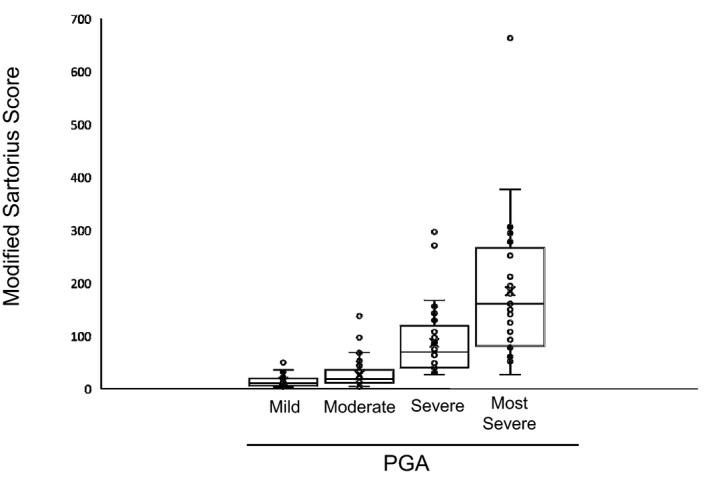
Correlation between the modified Sartorius score and Physician Global Assessment (PGA). The PGA score correlated with the modified Sartorius score (*P *< 0.001; Kruskal–Wallis test).

**Figure 2:**
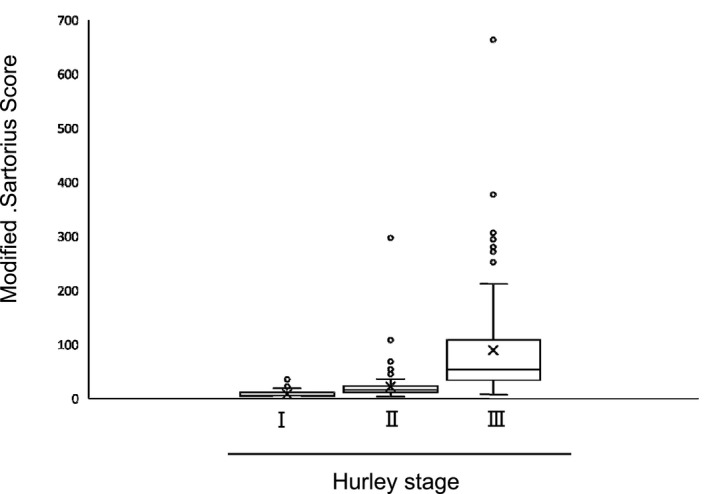
Correlation between modified Sartorius score and Hurley stage. Hurley stage correlated with the modified Sartorius score (*P* < 0.001; Kruskal–Wallis test).

We next examined the correlation between PGA score, Hurley stage and MSS. The numbers of patients classified as PGA score mild, moderate, severe and very severe were 100 (33.3%), 133 (44.3%), 34 (11.3%) and 29 (9.7%), respectively. The PGA scores for severe and very severe showed statistically significant correlation with MSS by Kruskal–Wallis test (*P* < 0.001; Table 3, Fig. 1). Multiple comparisons by Steel–Dwass test revealed significant difference between each group (*P *< 0.001, respectively; data not shown). There were 69 patients (23%) with Hurley stage I, 109 (36.3%) with stage II and 121 (40.3%) with stage III, and there was a statistically significant correlation with MSS by using the Kruskal–Wallis test (*P < *0.001; Table 3, Fig. 2). Multiple comparisons by Steel–Dwass test demonstrated a significant difference between each group (*P < *0.001, respectively; data not shown).

**Table 3:** Correlation between modified Sartorius score and disease severity

	Case (*n*)	Modified Sartorius score, median (IQR)
Hurley		
I	69	5 (4–10)
II	109	16 (11–22.5)
III	121	54 (34.5–102.5)
PGA		
Mild	100	10.5 (5–20)
Moderate	133	18.5 (11–35.5)
Severe	34	67 (40.75–113.5)
Most severe	29	161 (83–254)

IQR, interquartile range; PGA, Physician Global Assessment.

Although the difference was not statistically significant by the Mann–Whitney *U*‐test, there was a tendency that the scores of MSS were higher in patients with a family history (data not shown). We also investigated whether disease duration was related to disease severity, and found that patients with disease duration of more than 5 years showed higher PGA scores (χ^2^ = 28.688, *P < *0.001; Table 4).

**Table 4:** Association between PGA score and disease duration

Disease duration	Severity					
	Mild	Moderate	Severe	Most sever	Unknown	Total
<5 years	67	70	13	7	3	166
≥5 years	22	48	19	22	1	112
total	89	118	32	29	4	272

Hidradenitis suppurativa patients with disease duration of >5 years were significantly more severe (χ^2^ = 28.688, *P* < 0.001; χ^2^‐test). PGA, Physician Global Assessment.

Affected anatomical locations in Japanese HS patients are summarized in Table 5. Buttocks, axillae and groin were affected in 54.0%, 26.6% and 14.3%, respectively. The incidences of the lesions on the axillae, groin and buttock were 49 (22.4%), 19 (8.7%) and 162 (66.2%) of the 219 male patients, respectively, and 31 (38.3%), 24 (29.6%) and 17 (21.0%) of the 81 female patients, respectively. Interestingly, the presence or absence of lesions on the axillae, groin or buttocks was associated with sex (*P < *0.001, respectively; Table 5). The number of affected anatomical sites in each patient was also investigated based on the presence or absence of the lesion(s) on three anatomical sites: axillae, buttocks and groin (Table 6). The majority of the patients (188/300; 62.7%) had only one affected anatomical site. Patients suffering from lesions in two and three anatomical sites were 29 (9.7%) and 12 (4.0%) of the 300 patients, respectively. There was no relationship between the number of affected sites and sex (χ^2^ = 2.367, *P* = 0.102). Then, we examined whether there were any associations between the disease severity and affected region(s). Patients with lesion(s) in the axilla(e) had higher PGA scores (χ^2^ = 8.6378, *P* = 0.03452; Table 7). There were no significant differences for lesions on the buttocks (χ^2^ = 2.9012, *P* = 0.4071; data not shown) and groin (χ^2^ = 0.60793, *P = *0.8946; data not shown).

**Table 5:** Association between anatomical location and sex

		Presence of symptoms	No symptoms	Unknown	*P*
Axillae	M (*n* = 219)	49 (23.3%)	134 (61.2%)	37 (16.9%)	<0.001
	F (*n* = 81)	31 (38.3%)	32 (39.5%)	17 (21.0%)	
	Total (*n* = 300)	80 (26.6%)	166 (53.3%)	54 (18.0%)	
Groin	M (*n* = 219)	19 (8.7%)	200 (91.3%)	0	<0.001
	F (*n* = 81)	24 (29.6%)	57 (70.3%)	0	
	Total (*n* = 300)	43 (14.3%)	257 (85.7%)	0	
Buttocks	M (*n* = 219)	145 (66.2%)	74 (33.8%)	0	<0.001
	F (*n* = 81)	17 (21.0%)	64 (79.0%)	0	
	Total (*n* = 300)	162 (54.0%)	138 (46.0%)	0	

Breakdown of anatomical location of the lesions. In addition to total patient numbers, the number of males (M) and females (F) are also shown.

**Table 6:** Number of affected anatomical locations by sex

No. of affected sites	Total (*n* = 300)	Male (*n* = 219)	Female (*n* = 81)
1	190 (63.3%)	138 (63.0%)	52 (64.2%)
2	29 (9.7%)	20 (9.1%)	9 (11.1%)
3	12 (4.0%)	11 (5.0%)	1 (1.2%)
Others	69 (23.0%)	50 (22.8%)	19 (23.5%)

The numbers of affected anatomical sites are shown based on the presence or absence of the lesion(s) on three anatomical sites: axillae, buttocks and groin. Others include patients having lesions in the areas other than axillae, buttocks and groin.

**Table 7:** Association between anatomical location and PGA score

Symptom in axillae	Severity (PGA)					Total
	Mild	Moderate	Severe	Most severe	Unknown	
No	60	81	14	9	2	166
Yes	28	29	10	12	1	80
Unknown	12	23	10	8	1	54
Total	100	133	34	29	4	300

Patients with lesion(s) in the axilla(e) had higher Physician Global Assessment (PGA) score (χ^2^ = 8.6378; *P* = 0.03452; χ^2^‐test).

Table 8 shows the relationship between the Hurley stage and the treatments. One hundred and twenty‐two patients (40.6%) were administrated topical antimicrobials, among which nadifloxacin was the most frequently prescribed. On the other hand, oral antimicrobials were used in 231 (77.0%) patients. The most commonly used oral antimicrobials were cephem antimicrobials. Surgical excision was performed in 157 (52.3%) patients. Patients with Hurley stages Ⅱ and Ⅲ tended to undergo surgery (Table 8), although the difference was not statistically significant (χ^2^ = 7.800, *P* = 0.081).

**Table 8:** Association between disease severity and applied treatment

Treatment	Hurley stage		
	I	II	III
Topical antimicrobials	30	42	50
Oral antimicrobials	55	79	97
i.v. antimicrobials	17	19	32
Surgical resection	26	65	68

The relationship between severity and treatment was expressed as the number of patients. No significant correlation between disease severity and applied treatment was detected (χ^2^ = 7.800, *P* = 0.081).

## Discussion

The diagnostic criteria and severity classification used in this survey were prepared with reference to the European criteria proposed in the second congress organized by the Hidradenitis Suppurativa Foundation.^3^ While there were no histopathological components for diagnostic items in the European criteria,^3^ histopathological criteria were introduced into our diagnostic criteria as diagnostic aids to confirm the diagnosis by excluding other skin diseases because the concept of HS is still not well‐recognized in Japan. In addition, we think that histopathological examination is important to verify the presence or absence of SCC. Histopathological examinations were conducted in almost half of the 300 patients in this study. This may indicate that diagnostic histopathological evaluation is practical in Japan.

In the most recent European HS guidelines,^1^ the diagnostic criteria include a history of recurrent painful or purulent lesions more than twice/6 months. In our criteria, however, disease duration is not included. Considering that HS is a chronic condition, this issue should be taken into consideration when Japanese official diagnostic criteria are developed.

Familial HS has attracted attention in recent years.^6^, ^7^, ^8^ It was reported that 30–40% of HS patients in Europe have a family history.^7^ In the present study, only 12 (4%) of the 300 patients had a family history, indicating that the incidence of familial HS is low in Japan. This finding also confirms the result in another Japanese study that familial cases were only two (2%) of 100 HS cases.^2^ The very small number of patients with a family history may be caused by the fact that HS is not well understood in Japan, and therefore detailed family histories had not necessarily been obtained. Although there are few familial Japanese cases, a mutation in a gene encoding γ‐secretase subunit was found.^8^ It is known that familial HS patients tend to have more severe symptoms,^9^, ^10^ especially in patients with mutations in the genes encoding γ‐secretase subunits.^7^ Interestingly, in our study, disease severity tended to be higher in the 12 familial HS patients, although the difference did not reach statistical significance, probably because of the small number of patients with a family history.

Hidradenitis suppurativa is predominant in women in Western countries.^1^ However, we found that Japanese HS patients showed a male predominance, which was consistent with the previous Japanese survey.^2^ A similar result was also reported from Korea.^11^ In Turkey, which is located between Europe and Asia, HS is also more common in men.^12^ Various factors including genetic background behind the differential male : female ratios need to be further studied.

The relation between sex and the distribution of the lesions is also intriguing. The study in Minnesota, USA, showed no relation between sex and buttock symptom,^13^ while another report from Europe indicated that perianal region and buttocks were more commonly affected in male patients.^3^ Our study showed that buttock lesions were more frequently seen in males, which was in accordance with previous reports from Japan and Korea.^2^, ^14^ Thus, the predominance of buttock lesions in males may be a characteristic feature in HS patients of Japanese and Korean populations, or in Asian countries generally.

Unexpectedly, symptoms on the axillary fossae, but not on the buttocks, were correlated with disease severity in our study, although the reason for this result is currently uncertain. However, 18% of our patients lacked information about the axillary symptoms, which may reflect the fact that the concept of HS is still uncommon in Japan.

Western HS patients are at higher risk for obesity and it correlates with HS severity.^4^ We found tendencies for lower frequency of obesity compared with HS patients in Western countries.^1^ On the other hand, we found that diabetes mellitus was related to disease severity, which was not observed in Western populations.^15^, ^16^ Smoking is a well‐known exacerbating factor in HS,^13^ but it was not correlated with disease severity in Japanese patients. These results also suggest that the background factors for HS in Japan are different from those in Western countries. In our study, however, the risk of complications was not fully confirmed, probably because of the small number of HS patients.

Hidradenitis suppurativa may also develop secondary SCC.^17^ Prognosis is very poor in such cases.^17^ Even in Western countries with less male prevalence, SCC is four‐times more common in males, and often observed on the perineum and buttocks. After the onset of HS, it takes an average of 25 years to develop SCC.^17^ Another study reported similar results that the prevalence of SCC in HS is approximately 4.6% and is more common in men.^18^ During the period of this study, SCC was observed in only one patient (0.33%). This patient showed very severe HS with a family history and died of metastasis 2 years after the onset of SCC. The low frequency of the development of SCC in the current study may also be due to the lower degree of recognition in Japan, and HS may not be considered as a cause of SCC in the buttocks.

To determine the severity of HS, MSS has been widely used in the world.^1^ MSS was reported to correlate with the Hurley staging system,^4^ while there is no study that demonstrated the relationship between the PGA score and MSS. In our study, the correlation between MSS and the Hurley staging system was also found, and additionally MSS was correlated with PGA score. The median scores of MSS in this study were 10.5 (5–20), 18.5 (11–35.5), 67 (40.75–113.5) and 161 (83–254) in patients classified as mild, moderate, severe and most severe by PGA, respectively, verifying the usefulness of MSS to evaluate the severity of HS patients. Thus, our study has demonstrated for the first time that there is a highly significant correlation between PGA score and MSS.

In our study, the ratios of patients categorized as Hurley stage I, Ⅱ and Ⅲ were 23%, 36.3% and 40.3%, respectively, while those in Western countries were 68%, 28% and 4%, respectively,^1^ suggesting that Japanese patients may be more severe than Western patients. A high rate of severe forms in Japan was also reported by Kurokawa *et al.* ^2^ This discrepancy may be caused by some biases, because we surveyed only relatively large institutions, mostly university hospitals, which led to the increase in the number of cases of severe HS. In addition, by lower awareness of the concept of HS in Japan, many relatively mild types might have been underdiagnosed and not included in this study. Indeed, many respondents to our first questionnaire stated that they had no HS patients.

Hidradenitis suppurativa may be considered as an infectious disease. However, infections were able to be eliminated in this study because infection was an exclusion criterion. Nonetheless, many physicians used topical nadifloxacin, instead of topical clindamycin, the treatment recommended by the guidelines for HS.^1^ This implies that there is still a confusion with respect to the disease concept of HS in Japan. Correct information on the pathophysiology of HS needs to be widely recognized.

Limitations in our survey were that some information including the body mass index and Brinkman index were missing in the medical records, that we could not explore the treatment in detail, and that we were able to examine only past information due to the nature of cross‐sectional study.

In conclusion, this study showed actual pictures of HS in Japan, which differ from those seen in Western countries. However, prognosis, therapeutic outcome and QoL associated with the disease remain unclear. Further research is needed to better understand the detailed epidemiology of HS in Japan.

## Conflict of Interest

H. K. has received honoraria for speaking and consultancy from AbbVie, Eisai and Novartis, Sanofi and Mitsubishi Tanabe. H. F. has received honoraria for speaking and consultancy from AbbVie, Eisai, Novartis, Janssen Pharmaceutical, Marho, Taiho, Eli Lilly and Tanabe‐Mitubishi. T. T. has received honoraria for speaking and consultancy from AbbVie, Eisai, Novartis, Janssen Pharmaceutical, Marho, Taiho, Eli Lilly, Bristol‐Myers Squibb and Tanabe‐Mitubishi.

## References

[1] Zouboulis CC , Desai N , Emtestam L *et al.* European S1 guideline for the treatment of hidradenitis suppurativa/acne inversa. J Eur Acad Dermatol Venereol 2015; 29: 619–644. 25640693 10.1111/jdv.12966

[2] Kurokawa I , Hayashi N . Japan Acne Research Society. Questionnaire surveillance of hidradenitis suppurativa in. Japan. J Dermatol 2015; 42: 747–749. 10.1111/1346-8138.12881 25898994

[3] Revuz J . Hidradenitis suppurativa. J Eur Acad Dermatol Venereol. 2009; 23: 985–998. 19682181 10.1111/j.1468-3083.2009.03356.x

[4] Sartorius K , Emtestam L , Jemec GB *et al.* Objective scoring of hidradenitis suppurativa reflecting the role of tobacco smoking and obesity. Br J Dermatol 2009; 161: 831–839. 19438453 10.1111/j.1365-2133.2009.09198.x

[5] Hurley H . Axillary hyperhidrosis, apocrine bromhidrosis, hidradenitis suppurativa, and familial benign pemphigus: surgical approach. In: Roenigk RK, Roenigk HH, eds. Dermatologic Surgery. New York, NY: Marcel Dekker, 1989; 729–739.

[6] Von Der Werth JM , Williams HC , Raeburn JA . The clinical genetics of hidradenitis suppurativa revisited. Br J Dermatol 2000; 142: 947–953. 10809853 10.1046/j.1365-2133.2000.03476.x

[7] Pink AE , Simpson MA , Desai N *et al.* γ‐Secretase mutations in hidradenitis suppurativa: new insights into disease pathogenesis. J Invest Dermatol 2013; 133: 601–607. 23096707 10.1038/jid.2012.372

[8] Nomura Y , Nomura T , Sakai K *et al.* A novel splice site mutation in NCSTN underlies a Japanese family with hidradenitis suppurativa. Br J Dermatol 2013; 168: 206–209. 22834455 10.1111/j.1365-2133.2012.11174.x

[9] Schrader AM , Deckers IE , van der Zee HH , Boer J , Prens EP . Hidradenitis suppurativa: a retrospective study of 846 Dutch patients to identify factors associated with disease severity. J Am Acad Dermatol 2014; 71: 460–467. 24880664 10.1016/j.jaad.2014.04.001

[10] Deckers IE , van der Zee HH , Boer J , Prens EP . Correlation of early‐onset hidradenitis suppurativa with stronger genetic susceptibility and more widespread involvement. J Am Acad Dermatol 2015; 72: 485–488. 25582541 10.1016/j.jaad.2014.11.017

[11] Lee JH , Kwon HS , Jung HM , Kim GM , Bae JM . Prevalence and comorbidities associated with hidradenitis suppurativa in Korea: a nationwide population‐based study. J Eur Acad Dermatol Venereol 2018; 32: 1784–1790. 29761904 10.1111/jdv.15071

[12] Yüksel M , Basım P . Demographic and clinical features of hidradenitis suppurativa in Turkey. J Cutan Med Surg 2020; 24: 55–59. 31698918 10.1177/1203475419887732

[13] Vazquez BG , Alikhan A , Weaver AL , Wetter DA , Davis MD . Incidence of hidradenitis suppurativa and associated factors: a population‐based study of Olmsted County, Minnesota. J Invest Dermatol 2013; 133: 97–103. 22931916 10.1038/jid.2012.255 PMC3541436

[14] Yang JH , Moon J , Kye YC *et al.* Demographic and clinical features of hidradenitis suppurativa in Korea. J Dermatol 2018; 45: 1389–1395. 30294846 10.1111/1346-8138.14656

[15] Crowley JJ , Mekkes JR , Zouboulis CC *et al.* Association of hidradenitis suppurativa disease severity with increased risk for systemic comorbidities. Br J Dermatol 2014; 171: 1561–1565. 10.1111/bjd.13122 PMC4298243 24842009

[16] Revuz JE , Canoui‐Poitrine F , Wolkenstein P *et al.* Prevalence and factors associated with hidradenitis suppurativa: results from two case‐control studies. J Am Acad Dermatol 2008; 59: 596–601. 18674845 10.1016/j.jaad.2008.06.020

[17] Maclean GM , Coleman DJ . Three fatal cases of squamous cell carcinoma arising in chronic perineal hidradenitis suppurativa. Ann R Coll Surg Engl 2007; 89: 709–712. 17959012 10.1308/003588407X209392 PMC2121284

[18] Chapman S , Delgadillo D III , Barber C , Khachemoune A . Cutaneous squamous cell carcinoma complicating hidradenitis suppurativa: a review of the prevalence, pathogenesis, and treatment of this dreaded complication. Acta Dermatovenerol Alp Pannonica Adriat 2018; 27: 25–28. 29589641

